# Rho Inhibitor VX-210 in Acute Traumatic Subaxial Cervical Spinal Cord Injury: Design of the SPinal Cord Injury Rho INhibition InvestiGation (SPRING) Clinical Trial

**DOI:** 10.1089/neu.2017.5434

**Published:** 2018-05-01

**Authors:** Michael G. Fehlings, Kee D. Kim, Bizhan Aarabi, Marco Rizzo, Lisa M. Bond, Lisa McKerracher, Alexander R. Vaccaro, David O. Okonkwo

**Affiliations:** ^1^ Division of Neurosurgery and Spine Program, University of Toronto, Toronto, Ontario, Canada.; ^2^ Department of Neurological Surgery, University of California Davis School of Medicine, Sacramento, California.; ^3^ Department of Neurosurgery, University of Maryland School of Medicine, Baltimore, Maryland.; ^4^ Vertex Pharmaceuticals Incorporated, Boston, Massachusetts.; ^5^ BioAxone BioSciences, Inc, Cambridge, Massachusetts.; ^6^ Department of Orthopaedic Surgery, Sidney Kimmel Medical Center at Thomas Jefferson University, Philadelphia, Pennsylvania.; ^7^ Department of Neurological Surgery, University of Pittsburgh, Pittsburgh, Pennsylvania.

**Keywords:** motor recovery, Rho inhibition, spinal cord injury, SPRING trial, VX-210

## Abstract

Traumatic spinal cord injury (SCI) is associated with a lifetime of disability stemming from loss of motor, sensory, and autonomic functions; these losses, along with increased comorbid sequelae, negatively impact health outcomes and quality of life. Early decompression surgery post-SCI can enhance patient outcomes, but does not directly facilitate neural repair and regeneration. Currently, there are no U.S. Food and Drug Administration–approved pharmacological therapies to augment motor function and functional recovery in individuals with traumatic SCI. After an SCI, the enzyme, Rho, is activated by growth-inhibitory factors and regulates events that culminate in collapse of the neuronal growth cone, failure of axonal regeneration, and, ultimately, failure of motor and functional recovery. Inhibition of Rho activation is a potential treatment for injuries such as traumatic SCI. VX-210, an investigational agent, inhibits Rho. When administered extradurally after decompression (corpectomy or laminectomy) and stabilization surgery in a phase 1/2a study, VX-210 was well tolerated. Here, we describe the design of the SPRING trial, a multicenter, phase 2b/3, randomized, double-blind, placebo-controlled clinical trial to evaluate the efficacy and safety of VX-210 (NCT02669849). A subset of patients with acute traumatic cervical SCI is currently being enrolled in the United States and Canada. Medical, neurological, and functional changes are evaluated at 6 weeks and at 3, 6, and 12 months after VX-210 administration. Efficacy will be assessed by the primary outcome measure, change in upper extremity motor score at 6 months post-treatment, and by secondary outcomes that include question-based and task-based evaluations of functional recovery.

## Introduction

### Acute spinal cord injury

Current estimates indicate that 245,000–353,000 individuals who have suffered a spinal cord injury (SCI) are living in the United States, and approximately 40–54 new cases per year per million individuals occur.^1,2^ Common causes of SCI in the United States are motor vehicle accidents (38–42%), falls (31–33%), sports injuries (9–16%), and violence/other (9–18%).^1,3^ Worldwide, the estimated incidence of SCI ranges from 250,000–500,000 individuals per year,^4^ and approximately 2.5 million people live with an SCI.^3^

SCIs are often attributed to spinal cord compression and contusion that occur within a fraction of a second.^5^ Primary damage to the spinal cord is caused by the physical trauma, and further injury (secondary damage) is caused by downstream pathophysiological signaling cascades.^6^ This secondary damage is propagated through several mechanisms of action, including ischemia, excitotoxicity, cytotoxic and vasogenic edema, lipid peroxidation/radical formation, and inflammation.^7^ Further, axonal regeneration is impeded by growth-inhibiting factors that activate an intracellular master enzyme, Rho, leading to a cascade of events culminating in collapse of the neuronal growth cone.^8–12^ Both primary damage to neurons and axonal projections in the spinal cord and secondary damage post-injury result in loss of motor and sensory function in patients who experience an SCI; a severe traumatic lesion to the spinal cord can result in permanent paralysis below the segmental level of the injury.^13^

The independent daily activities of individuals who have suffered an SCI are often hampered; depending on the segmental level and injury severity, up to 24-h attendant care may be required for individuals with cervical SCI.^14,15^ Individuals with SCIs may also experience serious comorbidities that impact quality of life, including autonomic dysreflexia, bladder dysfunction, osteoporosis, heterotopic ossification, pressure sores, reduced immune function, chronic lifelong pain, and pulmonary and cardiovascular complications.^16–21^ Life expectancy of individuals with SCIs may be considerably shortened compared to age-matched controls from the general population.^1^ Because an SCI affects many aspects of life, even a partial restoration of motor function may improve patient autonomy and permit a higher quality of life.

### Treatment of spinal cord injury

A recognized standard of care for traumatic SCI is immediate immobilization, prevention of neurogenic shock, intubation, oxygenation, imaging evaluation, reduction, decompression, and stabilization. These measures, which help relieve direct pressure on the spinal cord and ischemic hypoxia, may reduce secondary damage.^5,22,23^ Although treatment of an acute SCI with decompression and stabilization surgery may provide clinical benefit to patients, this procedure does not directly facilitate axonal regeneration and repair.^3^ The primary goal of acute therapy for SCI is restoration of sufficient motor function to increase autonomy. Currently, there are no approved pharmacological treatments to augment motor function after an SCI. Therefore, SCI represents an extreme unmet medical need.

### VX-210, a Rho inhibitor

**FIG. 1.**
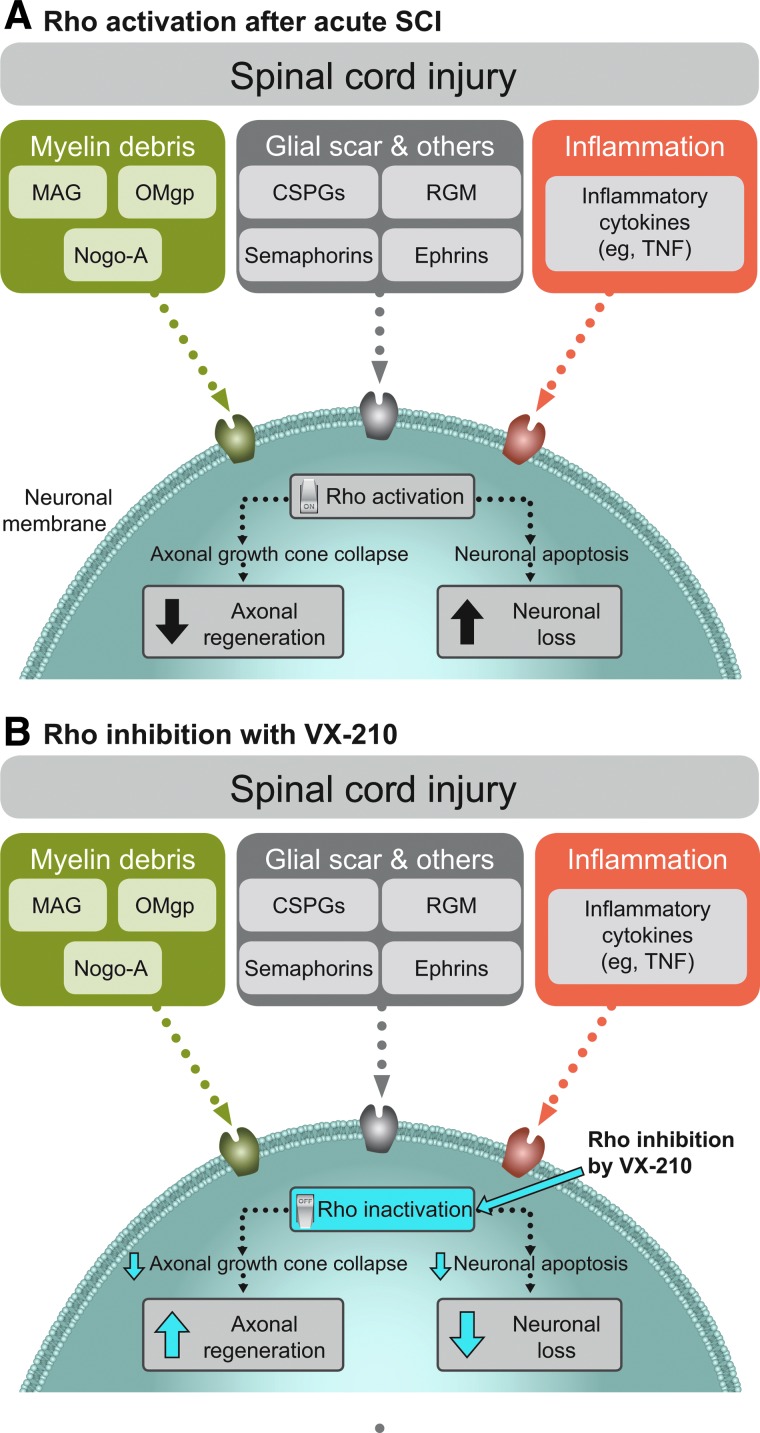
SCI-mediated Rho (**A**) activation and (**B**) inhibition by VX-210. CSPG, chondroitin sulfate proteoglycan; MAG, myelin-associated glycoprotein; Nogo-A, neurite outgrowth inhibitory protein A; OMgp, oligodendrocyte-myelin glycoprotein; RGM, repulsive guidance molecule; TNF, tumor necrosis factor.

The inability of patients to recover motor function after an SCI stems, in part, from the failure of neurons in the central nervous system (CNS) to regrow axons post-injury. In an ideal environment, axonal regeneration and sprouting might lead to the formation of new functional connections.^7^ After an acute SCI, however, axon regeneration is impeded by a number of growth-inhibitory proteins released by myelin debris, by the glial scar, and as a result of inflammation.^8,9,24^ These factors act in concert to activate the intracellular signaling molecule, Rho, by binding to and activating specific membrane receptors.^10,11^ Rho overactivation leads to a cascade of events culminating in the collapse of axonal growth cones^12^ and failure of injured axons to regenerate,^8,9^ as well as neuronal loss^25^ (Fig. 1). Inhibition of Rho kinase, a downstream effector of Rho, decreased neuronal injury in *in vitro* rat cortical neuron models.^26^ Thus, inhibition of Rho activity represents a potential treatment for CNS injuries such as SCI.

VX-210, formerly referred to as BA-210 or Cethrin, is a cell-permeable derivative of the bacterial enzyme, C3 transferase, that inhibits Rho activity through covalent modification and therefore has the potential to independently block Rho-mediated axonal growth cone collapse and inhibit neuronal apoptosis post-SCI.^25,27^ VX-210 is undergoing human investigation for its potential to augment motor function after acute cervical SCI.

### VX-210 administration

**FIG. 2.**
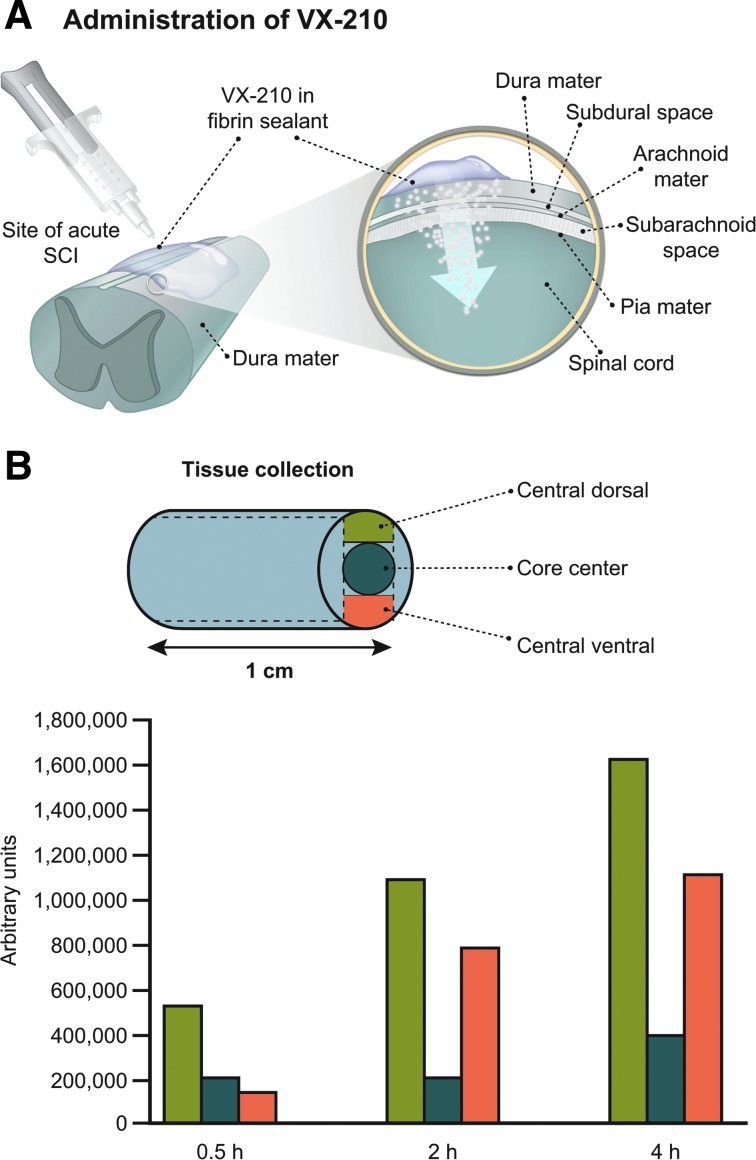
Penetration of VX-210 into spinal cord tissue. (**A**) Extradural administration and penetration into spinal cord tissue. (**B**) VX-210 relative penetration into spinal cord tissue in a post-mortem pig model after administration of a 1-mg dose. SCI, spinal cord injury.

VX-210 is administered topically in a fibrin sealant to the dura mater (extradural surface) of the spinal cord (Fig. 2A).^25,28^ Fibrin sealant is extensively used in spinal cord surgery for hemostasis and dural repairs. In rat and post-mortem porcine models, the concentration of VX-210 detected in spinal cord tissue after extradural application was dose dependent.^27,29^ Gradient levels of VX-210 were observed extending from the location of application within hours of extradural administration in both a rat SCI model and post-mortem pig spinal cord (Fig. 2B).^27,29^

### VX-210 in pre-clinical studies

Pre-clinical studies indicate that VX-210 has neuroregenerative and -protective effects and can promote functional recovery post-SCI. In rodent models of neuronal injury, treatment with VX-210 reversed the activation of Rho in spinal cord lesions, decreased secondary tissue damage and glial scarring at the injury site, and stimulated axon regeneration and plasticity in primary cortical neurons.^10,27,30–32^ In a mouse model of acute SCI, VX-210 administered onto the dura mater in fibrin sealant significantly improved Basso, Beattie, and Bresnahan open field locomotor rating scale scores compared to the control group (fibrin sealant alone) at 16 days post-injury when administered at the time of injury or 24 h post-injury.^27^

### VX-210 in early clinical evaluations: Phase 1/2a trial

The safety and tolerability of VX-210 were assessed in a multicenter, open-label, phase 1/2a dose-ranging trial (NCT00500812).^28,33,34^ In this trial, 48 patients with acute traumatic cervical (*n* = 16) and thoracic (*n* = 32) SCIs were enrolled at nine U.S. and Canadian sites. Patients were 16–70 years of age, with an American Spinal Injury Association Impairment Scale (AIS) grade A. Patients received a single dose of VX-210 (range, 0.3–9.0 mg) applied to the dura mater of the spinal cord during decompression surgery occurring ≤7 days post-injury and were followed and assessed for 1 year after treatment.^28^

The most frequent treatment-emergent adverse events (AEs; by organ system) were gastrointestinal disorders (nausea, constipation, and vomiting), general disorders (pyrexia, pain, and peripheral edema), infections, and psychiatric disorders (insomnia, anxiety, depression, and altered mood). Incidence of AEs was consistent across VX-210 dose levels, and no serious AEs (SAEs) were considered by investigators to be related to treatment. The results suggested improvement in motor strength in patients with cervical SCI compared to patients in natural history studies.^28^ In patients with thoracic SCI, the average recovery trajectory overlapped with that of past natural history studies.^5^

## Methods

VX-210 is under investigation as a potential therapy for acute cervical SCI in the phase 2b/3 **SP**inal Cord Injury **R**ho **IN**hibition Investi**G**ation (SPRING) trial. This article presents the design of the SPRING trial.

**FIG. 3.**
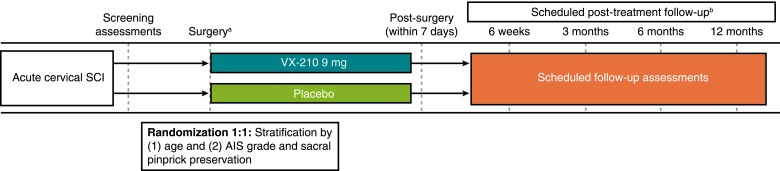
Study design of the phase 2b/3 SPRING trial. AIS, American Spinal Injury Association Impairment Scale; SCI, spinal cord injury. ^a^Application of VX-210/placebo occurs during decompression/stabilization surgery. ^b^All post-treatment follow-up dates occur within ±7 days.

SPRING is a multicenter, phase 2b/3, randomized, double-blind, placebo-controlled trial of VX-210 (NCT02669849), which is currently enrolling patients with acute traumatic cervical SCI (Fig. 3).^35^ Approximately 100 patients will be enrolled at about 45 sites in the United States and Canada (Table 1). The primary objective of this trial is to evaluate the efficacy and safety of VX-210 treatment. The primary endpoint, change from baseline in upper extremity motor score (UEMS) at 6 months, has been selected as a measure of clinically meaningful neurological improvement. Given that each of the muscles evaluated in the UEMS assessment is critical for daily function, small increases in UEMS can correspond to clinically meaningful improvements in functional recovery. Total motor score is not appropriate because 1) it assesses contraction strength of 10 key muscles in the upper and lower extremities on each side of the body and does not evaluate muscle groups by the potential for function or the functional value of an increase in score, and 2) the mechanism of action and target engagement would dictate a primary assessment focused on regeneration and sprouting local to the injury site. Secondary endpoints include examination of the effects of VX-210 on functional recovery by both question- and task-based assessments, change in AIS grade, and change in motor level.

**Table 1.** Clinical Trial Sites for the SPRING Trial in the United States and Canada

*State*	*City*	*Country*
Arizona	Tucson	USA
California	Los Angeles	USA
California	Orange	USA
California	Sacramento	USA
Connecticut	New Haven	USA
Florida	Jacksonville	USA
Florida	Tampa	USA
Georgia	Atlanta	USA
Georgia	Savannah	USA
Illinois	Chicago	USA
Iowa	Iowa City	USA
Kansas	Kansas City	USA
Maryland	Baltimore	USA
Massachusetts	Boston	USA
Michigan	Ann Arbor	USA
Minnesota	Minneapolis	USA
Mississippi	Jackson	USA
Missouri	St Louis	USA
New Jersey	Camden	USA
Ohio	Cleveland	USA
Ohio	Columbus	USA
Ohio	Toledo	USA
Pennsylvania	Hershey	USA
Pennsylvania	Philadelphia	USA
Pennsylvania	Pittsburgh	USA
Utah	Salt Lake City	USA
Washington	Seattle	USA
Alberta	Calgary	Canada
British Columbia	Vancouver	Canada
Nova Scotia	Halifax	Canada
Ontario	Ottawa	Canada
Ontario	Toronto	Canada
Québec	Montreal	Canada
Québec	Québec City	Canada
Saskatchewan	Saskatoon	Canada

Site and clinical trial information can be found at: www.vertexscitrial.com or www.clinicaltrials.gov (NCT02669849).

### Inclusion/exclusion criteria

Key inclusion and exclusion criteria for the study are summarized in Table 2. Eligible patients are between 14 and 75 years of age and have an acute traumatic cervical SCI. Patients must have an AIS grade of A or B; a motor level of C4, C5, C6, or C7 on each side as determined by a formal International Standards for Neurological Classification of Spinal Cord Injury (ISNCSCI) examination; and a screening UEMS of ≤16 points on each side (maximum total 32/50). Patients with AIS grade A and a C4 motor level on both sides must have ≥1 point of motor activity between C5 and T1 on at least one side; patients with AIS grade B and a C4 motor level on both sides must have ≥1 point of motor activity between C5 and C7 on at least one side. Patients must be scheduled to undergo spinal decompression/stabilization surgery commencing within 72 h after the initial injury, and computed tomography (CT) or magnetic resonance imaging (MRI) results must be consistent with the patient's neurological deficit. The subset of patients with C7 SCI added by protocol amendment was included to expand the range of eligible patients for the SPRING trial without compromising the primary analysis of change in UEMS.

**Table 2.** Eligibility Criteria: Phase 2b/3 SPRING Trial

	*Key inclusion criteria*	*Key exclusion criteria*
Patient characteristics	• 14–75 years, inclusive	• Body mass index ≥40 kg/m^2^ • Breastfeeding or pregnancy
Injury	• Acute traumatic cervical SCI	• Acute SCI from gunshot or penetrating/stab wound; nontraumatic SCI; brachial plexus injury; complete spinal cord transection; or multi-focal SCI
Neurological impairment	• Motor level of C4, C5, C6, or C7 on each side – Screening UEMS score must be ≤16 points on each side. – Patients with AIS grade A and a C4 motor level on both sides must have at least 1 point of motor activity between C5 and T1 on at least 1 side. – Patients with AIS grade B and a C4 motor level on both sides must have at least 1 point of motor activity between C5 and C7 on at least 1 side. • AIS grade A or B	• ≥1 upper extremity muscle group untestable during screening ISNCSCI examination • Unconsciousness or other mental impairment that precludes reliable ISNCSCI examination
Decompression/stabilization	• Scheduled and planned to undergo spinal cord decompression/stabilization surgery commencing within 72 h post-injury	• Inability to undergo decompression/stabilization surgery commencing within 72 h post-injury
Other	• CT or MRI consistent with neurological deficit	• Participation in any other clinical study for acute SCI without approval by the sponsor • Known immunodeficiency, including HIV or use of immunosuppressive or cancer chemotherapeutic drugs • History of an adverse reaction to a fibrin sealant or its components • Any significant medical or psychiatric comorbidities that would significantly increase the risk of study enrollment or significantly interfere with study outcomes or assessments, as judged by the investigator

AIS, American Spinal Injury Association Impairment Scale; CT, computed tomography; HIV, human immunodeficiency virus; ISNCSCI, International Standards for Neurological Classification of Spinal Cord Injury; MRI, magnetic resonance imaging; SCI, spinal cord injury; UEMS, upper extremity motor score.

Exclusion criteria include body mass index ≥40 kg/m^2^; acute SCI from a penetrating gunshot or stab wound, nontraumatic SCI, brachial plexus injury, complete spinal cord transection, or multifocal SCI; ≥1 upper extremity muscle group untestable during screening ISNCSCI examination; unconsciousness or mental impairment that precludes reliable screening ISNCSCI examination; inability to undergo decompression/stabilization surgery commencing within 72 h post-injury; known immunodeficiency, including human immunodeficiency virus (HIV) or the use of immunosuppressive or cancer chemotherapeutic drugs; breastfeeding or pregnancy; history of an adverse reaction to a fibrin sealant or its components; any significant medical or psychiatric comorbidities (e.g., neurological, cardiac, respiratory, hepatic, renal, bleeding/coagulation disorder, and active malignancy) that would significantly increase the risk of trial enrollment and/or significantly interfere with trial outcomes or assessments, as judged by the investigator; or participation in any other clinical study for acute SCI without approval by the study sponsor.

### Study design

In this double-blind, placebo-controlled study, each patient receives either a single 9-mg dose of VX-210 in fibrin sealant or a placebo (buffer solution) in fibrin sealant. The one-time treatment or placebo is administered by a surgeon directly onto the dura mater of the spinal cord at the site of injury during spinal cord decompression and internal stabilization surgery (Fig. 2). Patients are randomized to the 9-mg or placebo groups at a 1:1 ratio using an interactive Web or voice response system, with stratification by age (<30 vs. ≥30 years) and AIS grade (A vs. B with sacral pinprick preservation vs. B without sacral pinprick preservation). VX-210 and placebo are supplied in 2.0-mL glass vials, and the blinded study drug labeling complies with applicable local and national regulations. Each clinical site is provided with formulation preparation instructions and surgical guidelines to promote standardization of VX-210/placebo preparation and administration processes. All clinical site personnel are blinded throughout the study; unblinding of an individual patient's treatment by the investigator is limited to medical emergencies or urgent clinical situations in which knowledge of the patient's study treatment is necessary for clinical management. Follow-up assessments of recovery in VX-210-treated versus placebo-treated patients are conducted at 6 weeks and 3, 6, and 12 months post-treatment (Table 3). Medical, neurological, and functional changes are evaluated at pre-defined time points, and serum samples are collected for pharmacokinetic, biomarker, genetic, and immunological analyses. In the phase 1/2a trial, the 9-mg dose was the highest concentration tested, had a safe and tolerable safety profile,^28^ and was chosen to maximize target engagement.

**Table 3.** Schedule of Selected SPRING Trial Assessments

*Event/assessment*	*Screening*^a^	*Surgery*^b^	*Post-surgery*^c^	*6-week follow-up (±7 days)*^d^	*3-month follow-up (±7 days)*^d^	*6-month follow-up (±7 days)*^d^	*12-month follow-up (±7 days)*^d^	*Early termination*^d^	*Safety follow-up 28 (±3) days after treatment*^d,e^
AEs and past and concomitant medications and procedures	Continuous from signing of ICF through the last study visit								
Efficacy assessments									
ISNCSCI examination	X			X	X	X	X	X	X
SCIM III				X	X	X	X	X	X
GRASSP quantitative prehension						X		X	X
CUE-T						X		X	X
Follow-up questionnaire						X	X	X	X
SF-36				X		X	X	X	X
EQ-5D-5L						X	X	X	X
Hospitalizations	Continuous from signing of ICF through the last study visit								
Study drug administration									
VX-210 or placebo		X							
Pharmacokinetic assessments									
Serum samples for PK	X^f^	X^f^	X^f^						

^a^ Results of assessments performed as part of standard of care (except for ISNCSCI examination) within 72 h after initial injury and before signing of the ICF may be carried forward as screening results.

^b^ Surgery refers to the spinal decompression/stabilization surgery that commences within 72 h after initial injury, during which the study drug (VX-210 or placebo) is administered in a fibrin sealant.

^c^ Post-surgery assessments are performed within 7 days of completion of surgery.

^d^ Follow-up assessments of recovery are conducted at 6 weeks, 3 months, 6 months, and 12 months after treatment.

^e^ The safety follow-up visit is required in addition to the early termination visit only for patients who prematurely terminate from the study before day 28 after treatment. Patients who prematurely terminate from the study after day 28 are only required to complete the early termination visit.

^f^ Serum samples for PK analyses are collected at ≤72 h (before surgery) and at 3, 6, 12, 24, and 48 h after treatment and at the time of any SAE occurring within 3 days after treatment. The acceptable window for the post-treatment PK sampling time points is ±30 min.

AE, adverse event; CUE-T, capabilities of upper extremity test; EQ-5D-5L, 5-level European Quality of Life–5 Dimensions Questionnaire; GRASSP, graded redefined assessment of strength, sensibility and prehension; ICF, informed consent form; ISNCSCI, International Standards for Neurological Classification of Spinal Cord Injury; PK, pharmacokinetic; SAE, serious AE; SCIM, Spinal Cord Independence Measure; SF-36, Short Form 36 Health Survey.

### Study endpoints

The primary endpoint of this study is the change from baseline in UEMS at 6 months post-treatment. UEMS is a portion of the ISNCSCI neurological assessment that focuses on the hand and arm strength most relevant to individuals with a cervical SCI.^36^ Muscle contraction strength is graded in five key arm and hand muscle groups on each side of the body from 0 (total paralysis) to 5 ([normal] active movement, full range of motion against gravity, and full resistance in a functional muscle position expected from an otherwise unimpaired person), for a total possible UEMS of 50. Change from baseline to 6 months in UEMS for the VX-210 9-mg group will be compared to that of the placebo group for the primary evaluation of efficacy.

Secondary endpoints evaluating functional recovery include 1) the Spinal Cord Independence Measure III (SCIM III) self-care subscore (a question-based evaluation of a patient's ability to feed, dress, groom, and bathe independently on a daily basis) at 6 months post-treatment^37–39^; 2) the capabilities of upper extremity test (CUE-T) score (an evaluation of a patient's ability to perform specific functional movements or tasks with the arms and hands, such as grasping a pencil, pushing, or lifting a weight) at 6 months post-treatment^40,41^; and 3) the graded redefined assessment of strength, sensibility, and prehension (GRASSP) quantitative prehension score (an assessment of a patient's ability to perform specific functions with the arms, hands, and fingers, such as turning a key in a lock or pouring water in a cup) at 6 months post-treatment.^42–46^ Secondary endpoints evaluating neurological recovery include AIS grade conversion and motor level change from baseline to 6 months post-treatment, which are both derivatives of the ISNCSCI neurological assessment. Pharmacokinetic parameters of VX-210 will also be evaluated. The ISNCSCI, SCIM III, CUE-T, and GRASSP assessments are conducted by independent trained assessors. All efforts are made to use the same assessor for a given efficacy assessment for a given patient.

Safety evaluations include AEs, vital signs, electrocardiograms, clinical laboratory tests (i.e., serum chemistry, hematology, coagulation, and urinalysis), physical examinations, surgical site examinations, and immunogenicity measures. Safety and tolerability data will be reviewed by an independent data monitoring committee to ensure the safety of patients in the study.

### Interim analysis

An interim analysis will be conducted when 33% of enrolled patients have completed the 6-month follow-up visit, and the study may be stopped for futility depending on the results of this interim analysis. An independent data monitoring committee will conduct the review and make a recommendation to the study sponsor on the topic of futility.

### Statistical analysis

The null hypothesis to be tested is that the mean change from baseline in UEMS at 6 months post-treatment is the same for the 9-mg dose of VX-210 and placebo. This null hypothesis will be tested at a two-sided significance level (α = 0.05). Using a standard deviation of 6.0, if the 9-mg VX-210 group improves in UEMS by 4 points more than the placebo group (a clinically meaningful difference), the approximately 100 study patients (∼50 patients/group) will provide ≥80% power to detect a statistically significant treatment effect for the 9-mg VX-210 group compared to placebo. The primary analysis will include those who prematurely terminate before the 6-month follow-up.

To control the type I error rate, a hierarchical testing procedure will be used for the important efficacy endpoints. The first endpoint in the testing hierarchy will be the primary endpoint (change from baseline in UEMS at 6 months post-treatment). The second endpoint in the testing hierarchy will be the SCIM III self-care subscore at 6 months post-treatment. Treatment effects will be analyzed for each time point.

### Ethics and informed consent

The study is being conducted in accord with the current International Conference on Harmonisation Guideline for Good Clinical Practice (ICH GCP), which is consistent with the ethical principles founded in the Declaration of Helsinki, and in accord with local applicable laws and regulations. An institutional review board (IRB) or independent ethics committee (IEC) reviews all appropriate study documentation to safeguard the rights, safety, and well-being of the patients. The study is only conducted at sites where IRB/IEC approval has been obtained.

After the study has been fully explained, the patient (or a witness or legally appointed and authorized representative) signs and dates an informed consent form before study participation. The method of obtaining and documenting the informed consent and assent (if applicable) and the contents of the consent comply with the ICH GCP and all applicable laws and regulations.

## Conclusions

Substantial preclinical data support Rho overactivation as a key step in the inhibition of motor neurite outgrowth and promotion of neuronal apoptosis post-SCI. Inhibition of Rho thus represents a potential treatment for SCI. VX-210 is an investigational inhibitor of Rho. The phase 2b/3 SPRING trial (ClinicalTrials.gov identifier: NCT02669849) tests the hypothesis that VX-210 applied to the (extradural) dura mater diffuses into the spinal cord and augments motor recovery after acute cervical SCI. The SPRING trial is currently enrolling patients with acute cervical SCI throughout the United States and Canada.

## References

[1] National Spinal Cord Injury Statistical Center. Spinal cord injury facts and figures at a glance. 2017 Available at: https://www.nscisc.uab.edu Accessed 714, 2017

[2] Singh A., Tetreault L., Kalsi-Ryan S., Nouri A., and Fehlings M.G. (2014). Global prevalence and incidence of traumatic spinal cord injury. Clin. Epidemiol. 6, 309–331 25278785 10.2147/CLEP.S68889 PMC4179833

[3] Aarabi B., Sansur C.A., Ibrahimi D.M., Simard J.M., Hersh D.S., Le E., Diaz C., Massetti J., and Akhtar-Danesh N. (2017). Intramedullary lesion length on postoperative magnetic resonance imaging is a strong predictor of ASIA impairment scale grade conversion following decompressive surgery in cervical spinal cord injury. Neurosurgery 80, 610–620 28362913 10.1093/neuros/nyw053 PMC5748932

[4] World Health Organization. Spinal cord injury: fact sheet no. 384. 2013 Available at: http://www.who.int/mediacentre/factsheets/fs384/en/ Accessed 714, 2017

[5] Fehlings M.G., Vaccaro A., Wilson J.R., Singh A., Cadotte D.W., Harrop J.S, Aarabi B., Shaffrey C., Dvorak M., Fisher C., Arnold P., Massicotte E.M., Lewis S., and Rampersaud R. (2012). Early versus delayed decompression for traumatic cervical spinal cord injury: results of the surgical timing in acute spinal cord injury study (STASCIS). PLoS One 7, e32037 22384132 10.1371/journal.pone.0032037 PMC3285644

[6] Hausmann O.N. (2003). Post-traumatic inflammation following spinal cord injury. Spinal Cord 41, 369–378 12815368 10.1038/sj.sc.3101483

[7] Schwab J.M., Brechtel K., Mueller C.-A., Failli V., Kaps H.-P., Tuli S.K., and Schluesener H.J. (2006). Experimental strategies to promote spinal cord regeneration—an integrative perspective. Prog. Neurobiol. 78, 91–116 16487649 10.1016/j.pneurobio.2005.12.004

[8] Schwab M.E. (2004). Nogo and axon regeneration. Curr. Opin. Neurobiol. 14, 118–124 15018947 10.1016/j.conb.2004.01.004

[9] McKerracher L., and David S. (2004). Easing the brakes on spinal cord repair. Nat. Med. 10, 1052–1053 15459707 10.1038/nm1004-1052

[10] Dubreuil C.I., Winton M.J., and McKerracher L. (2003). Rho activation patterns after spinal cord injury and the role of activated Rho in apoptosis in the central nervous system. J. Cell Biol. 162, 233–243 12860969 10.1083/jcb.200301080 PMC2172802

[11] Madura T., Yamashita T., Kubo T., Fujitani M., Hosokawa K., and Tohyama M. (2004). Activation of Rho in the injured axons following spinal cord injury. EMBO Rep. 5, 412–417 15031718 10.1038/sj.embor.7400117 PMC1299028

[12] Shibata A., Wright M.V., David S., McKerracher L., Braun P.E., and Kater S.B. (1998). Unique responses of differentiating neuronal growth cones to inhibitory cues presented by oligodendrocytes. J. Cell Biol. 142, 191–202 9660873 10.1083/jcb.142.1.191 PMC2133022

[13] Dumont R.J., Okonkwo D.O., Verma S., Hurlbert R.J., Boulos P.T., Ellegala D.B., and Dumont A.S. (2001). Acute spinal cord injury, part I: pathophysiologic mechanisms. Clin. Neuropharmacol. 24, 254–264 11586110 10.1097/00002826-200109000-00002

[14] Steeves J.D., Lammertse D.P., Kramer J.L., Kleitman N., Kalsi-Ryan S., Jones L., Curt A., Blight A.R., and Anderson K.D. (2012). Outcome measures for acute/subacute cervical sensorimotor complete (AIS-A) spinal cord injury during a phase 2 clinical trial. Top. Spinal Cord Inj. Rehabil. 18, 1–14 23239927 10.1310/sci1801-1 PMC3519288

[15] Fawcett J.W., Curt A., Steeves J.D., Coleman W.P., Tuszynski M.H., Lammertse D., Bartlett P.F., Blight A.R., Dietz V., Ditunno J., Dobkin B.H., Havton L.A., Ellaway P.H., Fehlings M.G., Privat A., Grossman R., Guest J.D., Kleitman N., Nakamura M., Gaviria M., and Short D. (2007). Guidelines for the conduct of clinical trials for spinal cord injury as developed by the ICCP panel: spontaneous recovery after spinal cord injury and statistical power needed for therapeutic clinical trials. Spinal Cord 45, 190–205 17179973 10.1038/sj.sc.3102007

[16] Krassioukov A.V., Furlan J.C., and Fehlings M.G. (2003). Autonomic dysreflexia in acute spinal cord injury: an under-recognized clinical entity. J. Neurotrauma 20, 707–716 12965050 10.1089/089771503767869944

[17] Ackery A., Tator C., and Krassioukov A. (2004). A global perspective on spinal cord injury epidemiology. J. Neurotrauma 21, 1355–1370 15672627 10.1089/neu.2004.21.1355

[18] Cruse J.M., Lewis R.E., Dilioglou S., Roe D.L., Wallace W.F., and Chen R.S. (2000). Review of immune function, healing of pressure ulcers, and nutritional status in patients with spinal cord injury. J. Spinal Cord Med. 23, 129–135 10914354 10.1080/10790268.2000.11753520

[19] Krassioukov A.V., Furlan J.C., and Fehlings M.G. (2003). Medical co-morbidities, secondary complications, and mortality in elderly with acute spinal cord injury. J. Neurotrauma 20, 391–399 12866818 10.1089/089771503765172345

[20] Moore P.D., Gorgey A.S., Wade R.C., Khalil R.E., Lavis T.D., Khan R., and Adler R.A. (2016). Neuromuscular electrical stimulation and testosterone did not influence heterotopic ossification size after spinal cord injury: a case series. World J. Clin. Cases 4, 172–176 27458592 10.12998/wjcc.v4.i7.172 PMC4945587

[21] Zakrasek E.C., Nielson J.L., Kosarchuk J.J., Crew J.D., Ferguson A.R., and McKenna S.L. (2017). Pulmonary outcomes following specialized respiratory management for acute cervical spinal cord injury: a retrospective analysis. Spinal Cord 55, 559–565 28220822 10.1038/sc.2017.10 PMC5457341

[22] Bonner S., and Smith C. (2013). Initial management of acute spinal cord injury. Contin. Educ. Anaesth. Crit. Care Pain 13, 224–231

[23] Furlan J.C., Noonan V., Cadotte D.W., and Fehlings M.G. (2011). Timing of decompressive surgery of spinal cord after traumatic spinal cord injury: an evidence-based examination of pre-clinical and clinical studies. J. Neurotrauma 28, 1371–1399 20001726 10.1089/neu.2009.1147 PMC3143409

[24] Filbin M.T. (2003). Myelin-associated inhibitors of axonal regeneration in the adult mammalian CNS. Nat. Rev. Neurosci. 4, 703–713 12951563 10.1038/nrn1195

[25] McKerracher L., and Guertin P. (2013). Rho as a target to promote repair: translation to clinical studies with Cethrin. Curr. Pharm. Des. 19, 4400–4410 23360272 10.2174/1381612811319240007

[26] Hemphill M.A., Dabiri B.E., Gabriele S., Kerscher L., Franck C., Goss J.A., Alford P.W., and Parker K.K. (2011). A possible role for integrin signaling in diffuse axonal injury. PLoS One 6, e22899 21799943 10.1371/journal.pone.0022899 PMC3142195

[27] Lord-Fontaine S., Yang F., Diep Q., Dergham P., Munzer S., Tremblay P., and McKerracher L. (2008). Local inhibition of Rho signaling by cell-permeable recombinant protein BA-210 prevents secondary damage and promotes functional recovery following acute spinal cord injury. J. Neurotrauma 25, 1309–1322 19061375 10.1089/neu.2008.0613

[28] Fehlings M.G., Theodore N., Harrop J., Maurais G., Kuntz C., Shaffrey C.I., Kwon B.K., Chapman J., Yee A., Tighe A., and McKerracher L. (2011). A phase I/IIa clinical trial of a recombinant Rho protein antagonist in acute spinal cord injury. J. Neurotrauma 28, 787–796 21381984 10.1089/neu.2011.1765

[29] Fehlings M.G., Bond L.M., and Rizzo M. (2016). Rho inhibitor VX-210 in acute traumatic cervical spinal cord injury: design of the phase 2b/3 SPinal cord injury Rho INhibition investiGation (SPRING) trial. Poster presented at the 34th Annual Symposium of the National Neurotrauma Society, 626–29, Lexington, KY

[30] Dergham P., Ellezam B., Essagian C., Avedissian H., Lubell W.D., and McKerracher L. (2002). Rho signaling pathway targeted to promote spinal cord repair. J. Neurosci. 22, 6570–6577 12151536 10.1523/JNEUROSCI.22-15-06570.2002 PMC6758168

[31] Fournier A.E., Takizawa B.T., and Strittmatter S.M. (2003). Rho kinase inhibition enhances axonal regeneration in the injured CNS. J. Neurosci. 23, 1416–1423 12598630 10.1523/JNEUROSCI.23-04-01416.2003 PMC6742251

[32] Shearer M.C., Niclou S.P., Brown D., Asher R.A., Holtmaat A.J., Levine J.M., Verhaagen J., and Fawcett J.W. (2003). The astrocyte/meningeal cell interface is a barrier to neurite outgrowth which can be overcome by manipulation of inhibitory molecules or axonal signalling pathways. Mol. Cell. Neurosci. 24, 913–925 14697658 10.1016/j.mcn.2003.09.004

[33] Bond L.M., and McKerracher L. (2014). Cervical spinal cord injury: tailoring clinical trial endpoints to reflect meaningful functional improvements. Neural Regen. Res. 9, 1493–1497 25317162 10.4103/1673-5374.139470 PMC4192962

[34] McKerracher L., and Anderson K.D. (2013). Analysis of recruitment and outcomes in the phase I/IIa Cethrin clinical trial for acute spinal cord injury. J. Neurotrauma 30, 1795–1804 23844986 10.1089/neu.2013.2909

[35] Study to assess the efficacy and safety of VX-210 in subjects with acute traumatic cervical spinal cord injury. 2016 Available at: https://clinicaltrials.gov/ct2/show/NCT02669849 Accessed 714, 2017

[36] Steeves J.D., Lammertse D., Curt A., Fawcett J.W., Tuszynski M.H., Ditunno J.F., Ellaway P.H., Fehlings M.G., Guest J.D., Kleitman N., Bartlett P.F., Blight A.R., Dietz V., Dobkin B.H., Grossman R., Short D., Nakamura M., Coleman W.P., Gaviria M., and Privat A. (2007). Guidelines for the conduct of clinical trials for spinal cord injury (SCI) as developed by the ICCP panel: clinical trial outcome measures. Spinal Cord 45, 206–221 17179972 10.1038/sj.sc.3102008

[37] Itzkovich M., Gelernter I., Biering-Sorensen F., Weeks C., Laramee M.T., Craven B.C., Tonack M., Hitzig S.L., Glaser E., Zeilig G., Aito S., Scivoletto G., Mecci M., Chadwick R.J., El Masry W.S., Osman A., Glass C.A., Silva P., Soni B.M., Gardner B.P., Savic G., Bergström E.M., Bluvshtein V., Ronen J., and Catz A. (2007). The Spinal Cord Independence Measure (SCIM) version III: reliability and validity in a multi-center international study. Disabil. Rehabil. 29, 1926–1933 17852230 10.1080/09638280601046302

[38] Anderson K.D., Acuff M.E., Arp B.G., Backus D., Chun S., Fisher K., Fjerstad J.E., Graves D.E., Greenwald K., Groah S.L., Harkema S.J, Horton J.A. III, Huang M.-N., Jennings M., Kelley K.S., Kessler S.M., Kirshblum S., Koltenuk S., Linke M., Ljungerg I., Nagy J., Nicolini L., Roach M.J., Salles S., Scelza W.M., Read M.S., Reeves R.K., Scott M.D., Tansey K.E., Theis J.L., Tolfo C.Z., Whitney M., Williams C.D., Winter C.M., and Zanca J.M. (2011). United States (US) multi-center study to assess the validity and reliability of the Spinal Cord Independence Measure (SCIM III). Spinal Cord 49, 880–885 21445081 10.1038/sc.2011.20

[39] Bluvshtein V., Front L., Itzkovich M., Aidinoff E., Gelernter I., Hart J., Biering-Soerensen F., Weeks C., Laramee M.T., Craven C., Hitzig S.L., Glaser E., Zeilig G., Aito S., Scivoletto G., Mecci M., Chadwick R.J., El Masry W.S., Osman A., Glass C.A., Silva P., Soni B.M., Gardner B.P., Savic G., Bergström G., and Catz A. (2011). SCIM III is reliable and valid in a separate analysis for traumatic spinal cord lesions. Spinal Cord 49, 292–296 20820178 10.1038/sc.2010.111

[40] Marino R.J., Kern S.B., Leiby B., Schmidt-Read M., and Mulcahey M.J. (2015). Reliability and validity of the capabilities of upper extremit test (CUE-T) in subjects with chronic spinal cord injury. J. Spinal Cord Med. 38, 498–504 25297342 10.1179/2045772314Y.0000000272 PMC4612205

[41] Marino R.J., Patrick M., Albright W., Leiby B.E., Mulcahey M.J., Schmidt-Read M., and Kern S.B. (2012). Development of an objective test of upper-limb function in tetraplegia. Am. J. Phys. Med. Rehabil. 91, 478–486 22469875 10.1097/PHM.0b013e31824fa6cc

[42] Velstra I.-M., Curt A., Frotzler A., Abel R., Kalsi-Ryan S., Rietman J.S., and Bolliger M. (2015). Changes in strength, sensation, and prehension in acute cervical spinal cord injury: European multicenter responsiveness study of the GRASSP. Neurorehabil. Neural Repair 29, 755–766 25567122 10.1177/1545968314565466

[43] Kalsi-Ryan S., Curt A., Fehlings M.G., and Verrier M.C. (2009). Assessment of the hand in tetraplegia using the graded redefined assessment of strength, sensibility and prehension (GRASSP): impairment versus function. Top. Spinal Cord Inj. Rehabil. 14, 34–46

[44] Velstra I.-M., Bolliger M., Tanadini L.G., Baumberger M., Abel R., Rietman J.S., and Curt A. (2014). Prediction and stratification of upper limb function and self-care in acute cervival spinal cord injury with the graded redefined assessment of strength, sensibility, and prehension (GRASSP). Neurorehabil. Neural Repair 28, 632–642 24566986 10.1177/1545968314521695

[45] Kalsi-Ryan S., Curt A., Verrier M.C., and Fehlings M.G. (2012). Development of the graded redefined assessment of strength, sensibility and prehension (GRASSP): reviewing measurement specific to the upper limb in tetraplegia. J. Neurosurg. Spine 17, 1 Suppl., 65–76 22985372 10.3171/2012.6.AOSPINE1258

[46] Kalsi-Ryan S., Beaton D., Curt A., Popovic M.R., Verrier M.C., and Fehlings M.G. (2014). Outcome of the upper limb in cervical spinal cord injury: profiles of recovery and insights for clinical studies. J. Spinal Cord Med. 37, 503–510 25229734 10.1179/2045772314Y.0000000252 PMC4166185

